# Supplementary material for: Structural mechanism of TRPV3 channel inhibition by the anesthetic dyclonine
Source: Nat Commun. 2022 May 19;13:2795. doi: 10.1038/s41467-022-30537-8 (PMC9120478; doi:10.1038/s41467-022-30537-8)
Supplement: Supplementary file 1 — Supplementary Information [file 41467_2022_30537_MOESM1_ESM.pdf]

Supplementary Information

Structural mechanism of TRPV3 channel inhibition by the anesthetic dyclonine

Arthur Neuberger[1], Kirill D Nadezhdin[1], Alexander I Sobolevsky[1,*]

[1]Department of Biochemistry and Molecular Biophysics, Columbia University, New York, NY, USA

*Corresponding author. Tel: +1 2123054249; E-mail: as4005@cumc.columbia.edu

This PDF file includes:

Supplementary Figures 1-2

Supplementary Table 1

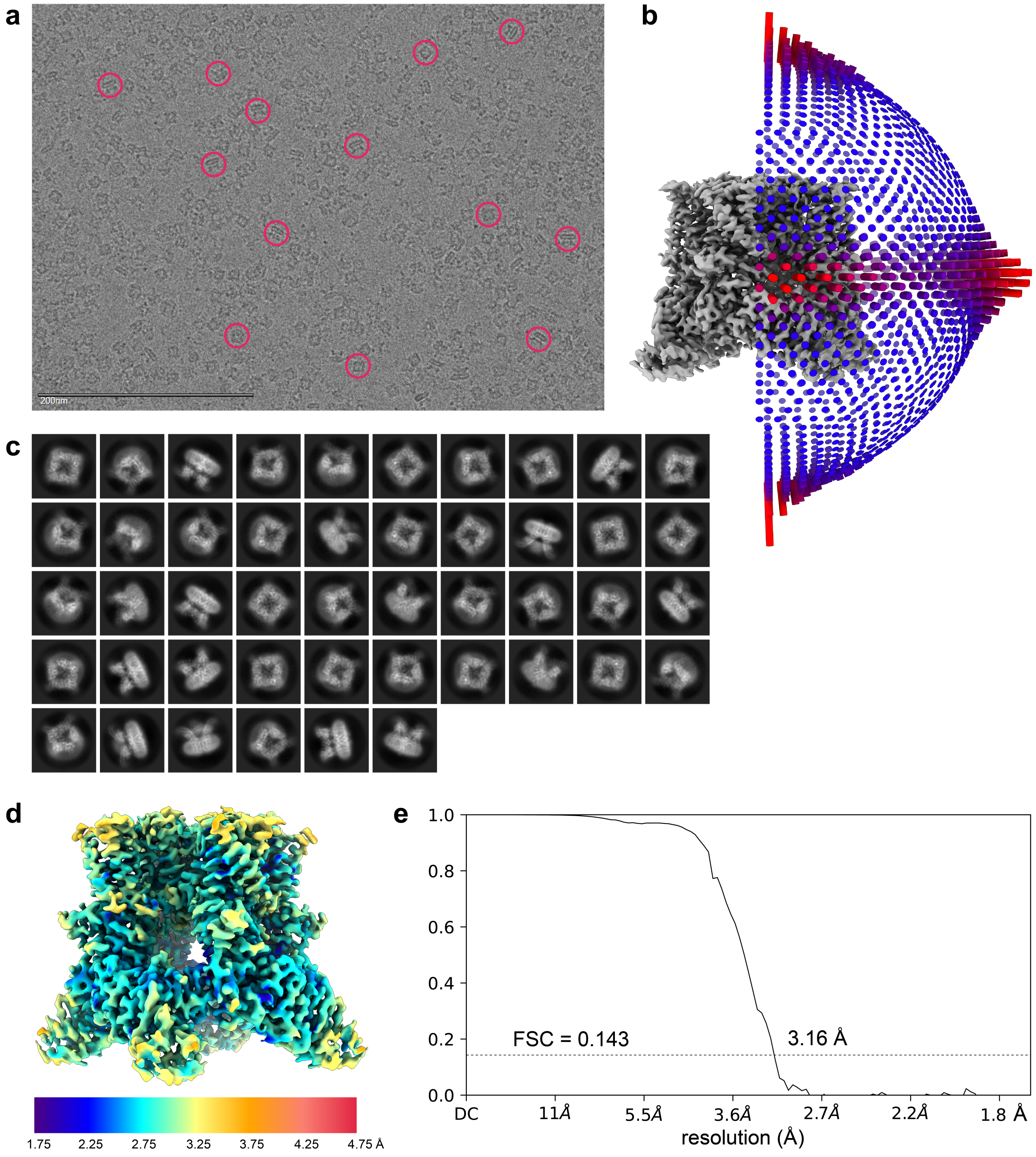

Supplementary Fig. 1. Overview of cryo-EM data for TRPV3$_{Dyc}$. a, Representative micrograph with example particles circled in pink. **b**, Euler angle distribution of particles contributing to the final reconstruction with larger red cylinders representing orientations comprising more particles. **c**, Reference-free 2D class averages. **d**, Local resolution presented as coloring of the TRPV3$_{Dyc}$ cryo-EM map. **e**, FSC curve.

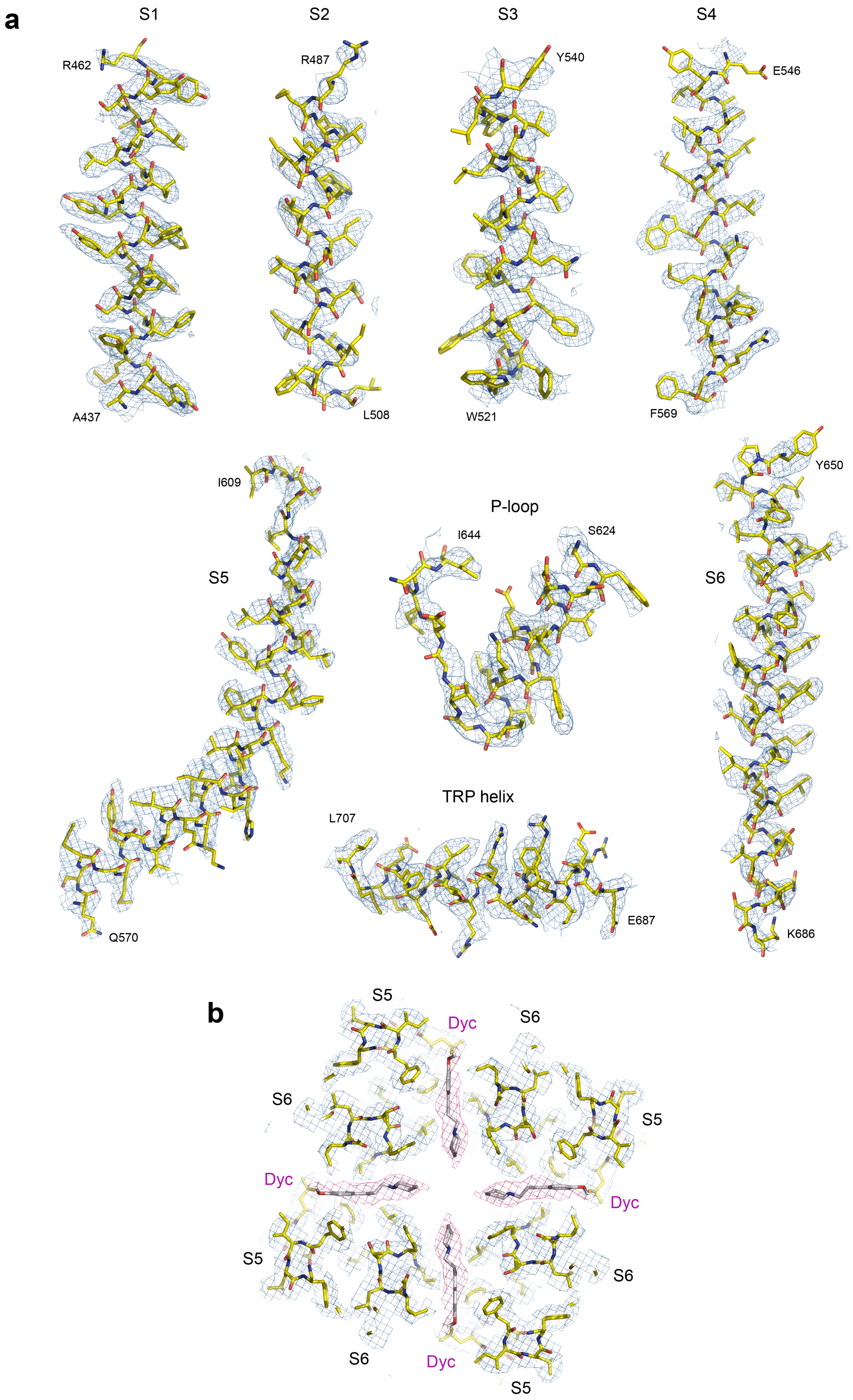

Supplementary Fig. 2. Cryo-EM density of TRPV3$_{\mathbf{Dyc}}$. a, Fragments of TRPV3$_{Dyc}$ cryo-EM map (blue mesh) for the membrane segments and TRP helix. **b**, The central slice of the map at the plane of dyclonine molecules. Density that corresponds to dyclonine molecules is shown as pink mesh.

Supplementary Table 1. Cryo-EM data collection, refinement and validation statistics

**Structure**	TRPV3$_{Dyc}$
EMDB accession code	EMD-26488
PDB accession code	7UGG
**Data collection and processing**	
Magnification	105,000x
Voltage (kV)	300
Electron exposure ($e^{-}Å^{-2}$)	60
Defocus range (μm)	−0.8 to −2.0
Reported pixel size (Å)	0.86
Exposures (no.)	6,856
**Processing software**	
Platform software for particle picking	cryoSPARC v3.1
Motion correction	cryoSPARC v3.1
CTF estimation	cryoSPARC v3.1
Software for 2D/3D class. & refinements	cryoSPARC v3.1
Symmetry imposed	C4
Initial particle images (no.)	4,985,077
Final particle images (no.)	87,876
Map resolution (Å)	3.16
FSC 0.143	
**Refinement**	
Initial models used (PDB code)	This study
Model resolution (Å)	3.16
FSC threshold	0.143
Map sharpening *B* factor ($Å^2$)	−132.9
**Model composition**	
Non-hydrogen atoms	21,670
Protein residues	2480
Ligands	30
Water	124
B factors ($Å^2$)	
Protein	63.11
Ligands	17.75
R.m.s. deviations	
Bond lengths (Å)	0.008
Bond angles (°)	1.241
**Validation**	
MolProbity score	1.60
Clash score, all atoms	3.36
Poor rotamers (%)	0.54
Ramachandran plot	
Favoured (%)	92.51
Allowed (%)	7.17
Disallowed (%)	0.33
